# The incidence of occult and missed surgical neck fractures in patients with isolated greater tuberosity fracture of the proximal humerus

**DOI:** 10.1186/s12891-019-2810-y

**Published:** 2019-10-27

**Authors:** Leo Shaw, Chih-Kai Hong, Fa-Chuan Kuan, Cheng-Li Lin, Ping-Hui Wang, Wei-Ren Su

**Affiliations:** 1 Department of Medical Education, Taichung Veteran’s General Hospital, Taichung, Taiwan; 2 Department of Orthopaedic Surgery, National Cheng Kung University Hospital, College of Medicine, National Cheng Kung University, No.138, Sheng-Li Road, Tainan City, Taiwan 70428; 3 Department of Orthopaedic Surgery, Chi-Mei Medical Center, Tainan, Taiwan; 4 Skeleton Materials and Bio-compatibility Core Lab, Research Center of Clinical Medicine, National Cheng Kung University Hospital, College of Medicine, National Cheng Kung University, Tainan, Taiwan

**Keywords:** Proximal humerus, Greater tuberosity fracture, Occult surgical neck fracture

## Abstract

**Background:**

Occult and missed surgical neck fractures can be found in patients diagnosed with isolated greater tuberosity (GT) fracture during the follow up period. The purpose of this study was to retrospectively assess the incidence rate of occult and missed surgical neck fractures in those initially diagnosed with isolated GT fracture.

**Methods:**

Records of patients diagnosed as having an isolated GT fracture were retrieved from a database in a medical center. Two senior orthopedic surgeons blindly reviewed all images of these patients three times to classify GT fracture types (split, avulsion and depression types), and recorded any surgical neck fractures found. Then a meeting was help to confirm the fracture types and presence of surgical neck fracture.

**Results:**

Occult surgical neck fractures were found in 5 out of 68 (7.4%) patients, whereas missed surgical neck fractures were found in 3 out of 68 (4.4%) patients. In total, 32 patients had split type GT fracture, 32 had avulsion type and 4 had depression type. For those with occult surgical neck fractures, 7 had the split type GT fracture, while the remaining one had the avulsion type. Although the proportion of occult surgical neck fracture was higher in the split-type GT fracture (21.9%) than in the avulsion-type GT fracture (3.1%), the difference was not statistically significant (*p* = 0.056).

**Conclusion:**

Occult humeral surgical neck fractures occurred in 7.4% of isolated greater tuberosity fractures after re-evaluation, while missed humeral surgical neck fractures occurred in 4.4%.

## Background

Proximal humeral fractures are the third most common type of osteoporotic fracture, after wrist and hip fractures [1]. Displaced three-part fractures, involving the greater tuberosity (GT) and surgical neck, are considered severe injuries in the spectrum of proximal humeral fractures, which are commonly treated surgically except for elderly patients, low-demand patients, and those with significant medical comorbidities [2, 3].

Isolated GT fracture represents approximately 20% of proximal humeral fractures, which occurs as the result of specific trauma mechanisms [4]. Diagnosis of a displaced GT fracture is obviously based on the history, physical examination, and radiographic finding; however, a concomitant surgical neck fracture may be missed or ignored if it is undisplaced. Given that the clinical presentations of the so-called occult surgical neck fractures are often indistinguishable when comparing to a patient with an isolated GT fracture, such diagnoses are often delayed until obvious displacement or healing evidence of surgical neck fracture is found in the follow-up radiography. However, delayed diagnosis of a coexisting surgical neck fracture might lead to poor outcome of the patient because of insufficient activity restriction and improper rehabilitation protocol. Previous reports have suggested that surgical neck fracture may lead to axis deviation, impaction, displacement, and loss of reduction, which could subsequently result in a substantial decrease in the quality of life [5, 6]. Moreover, the treatment selections for isolated GT fractures and GT associated with surgical neck fractures are different; therefore, acquiring more information on the existence of occult surgical neck fracture would help in clinical practice.

To our knowledge, however, no published study has investigated the incidence of occult or missed surgical neck fracture with displaced GT fracture. The purpose of this study was to retrospectively assess the incidence rate of occult surgical neck fracture in those who were diagnosed with isolated GT fracture at the initial visit and clarify the relationship between the characteristics of GT fractures and concomitant occult surgical neck fractures.

## Methods

After institutional review board approval, a database of electronic medical recordings was searched for patients who were diagnosed as having an isolated GT fracture between January 2002 and December 2015 at the authors’ hospital. Patients more than 18 years of age who were diagnosed as isolated GT fracture by a senior orthopedic resident and a radiologist during their first visit after trauma were included; the minimal follow-up duration for these patients was 4 weeks. Patients with pathological fractures, evidence of previous bony injuries or co-existed fracture of the proximal humerus were excluded.

**Fig. 1:**
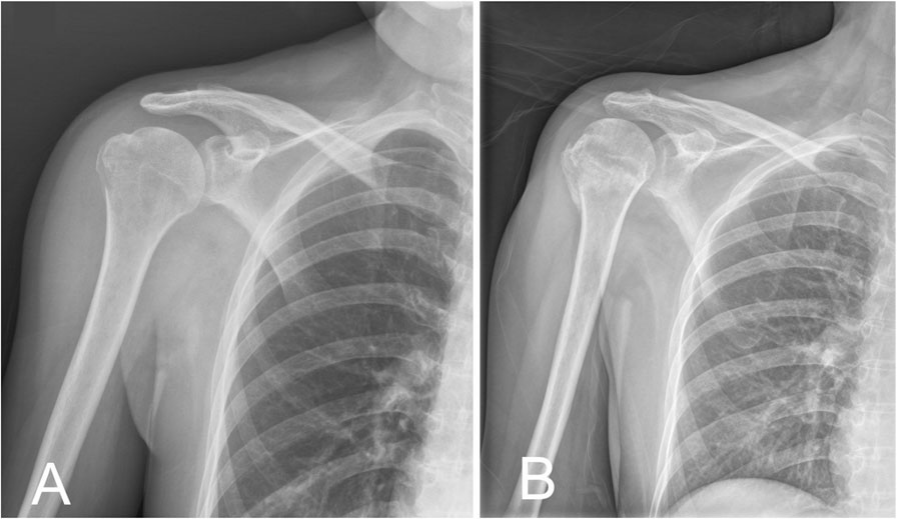
Radiographic findings of occult surgical neck fracture in a patient. **a** Isolated GT fracture was diagnosed from the radiograph on day 1. **b** On day 13, radiopacity at the surgical neck implied periosteal new bone formation, which indicated the existence of the occult surgical neck fracture

All patients with weakened abduction and external rotation received routine radiographic evaluation for injuries of proximal humerus if there was no suspicion of other proximal humeral fractures. The routine radiographic evaluation included anteroposterior views of internal and external rotation of injured shoulder. Further image evaluation was performed if the reduction technique was done for fracture or dislocation. Radiographs in the initial status were evaluated by senior orthopedic residents with the use of the Picture Archiving and Communication System (PACS – McKesson Corp., Montreal, Canada) (Fig. 1).

**Fig. 2:**
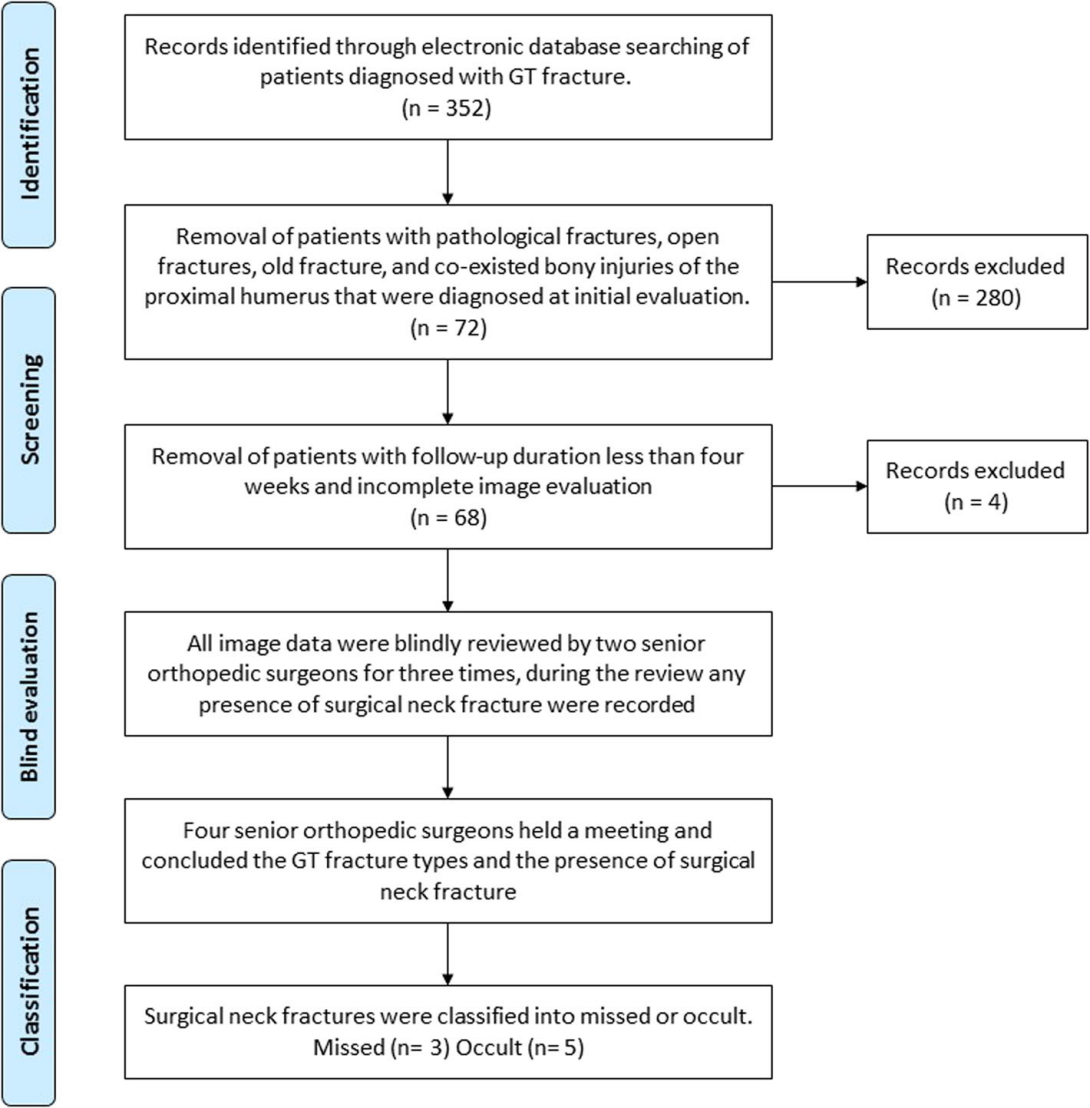
Review process of all patients diagnosed with greater tuberosity fracture

During the study period, 352 patients who were diagnosed as GT fracture was selected for this study. After reviewing the patients’ records, 280 patients were excluded because they had previous bony injuries or other co-existed fractures of proximal humerus that were diagnosed by the radiologists. In the remaining 72 patients, 4 patients were excluded because the follow-up durations were less than 4 weeks. After the identification and screening process, 68 patients (29 males and 39 females) with a mean age of 42 (26–58 years) were included in our study. The review process was illustrated in Fig. 2.

**Fig. 3:**
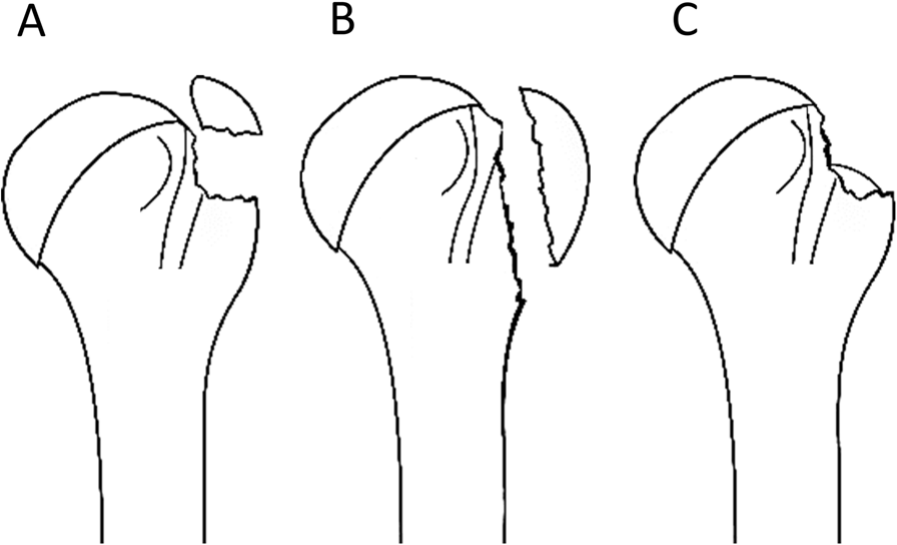
Illustration of the (**a**) avulsion-type, (**b**) split-type, and (**c**) depression-type GT fractures according to the classification proposed by Mutch et al. [7]

During the review process, two senior orthopedic surgeons (C-L L and W-R S) blindly re-evaluated the radiographs from 68 patients three times. The GT fractures were classified into split, avulsion or depression type (Fig. 3) according to Mutch et al. [7]. If a surgical neck fracture could be found from the evaluation, the radiograph was especially recorded. The angulations of the surgical neck fractures were evaluated by measuring the head-shaft angle in the anteroposterior radiographs [3]. After the blind evaluation, intra- and inter-observer reliability were assessed by calculating intra-class correlation coefficients (ICCs). A meeting was held with four senior orthopedic surgeons to conclude the GT fracture types and confirm the presence of surgical neck fracture.

After the review process, basic demographic variables of the patients, including age, gender, side of injury, and the treatment methods, were recorded. The causes of injury were classified into two categories, high energy injury or low energy. High energy injuries included motor-vehicle accidents and falls from heights above 2 m, where areas low energy included falls from heights less than 2 m and sport related injury.

### Definition of “missed” and “occult” surgical neck fractures

If a surgical neck fracture that was not found initially in clinical practice could be found from the retrospective image review, it would be considered as a “missed” surgical neck fracture. If a surgical neck fracture still could not be found from the retrospective image review and could only be found from other radiographs during the follow-up period, it would be considered as a “occult” surgical neck fracture.

### Statistics

Statistical analysis was performed with SPSS 17.0 software (SPSS Inc., Chicago, IL, USA). The Chi-square test was applied to compare the incidence rate of occult surgical neck fracture among the different types of GT fractures. A *p* < 0.05 was considered as statistically significant.

## Results

**Fig. 4:**
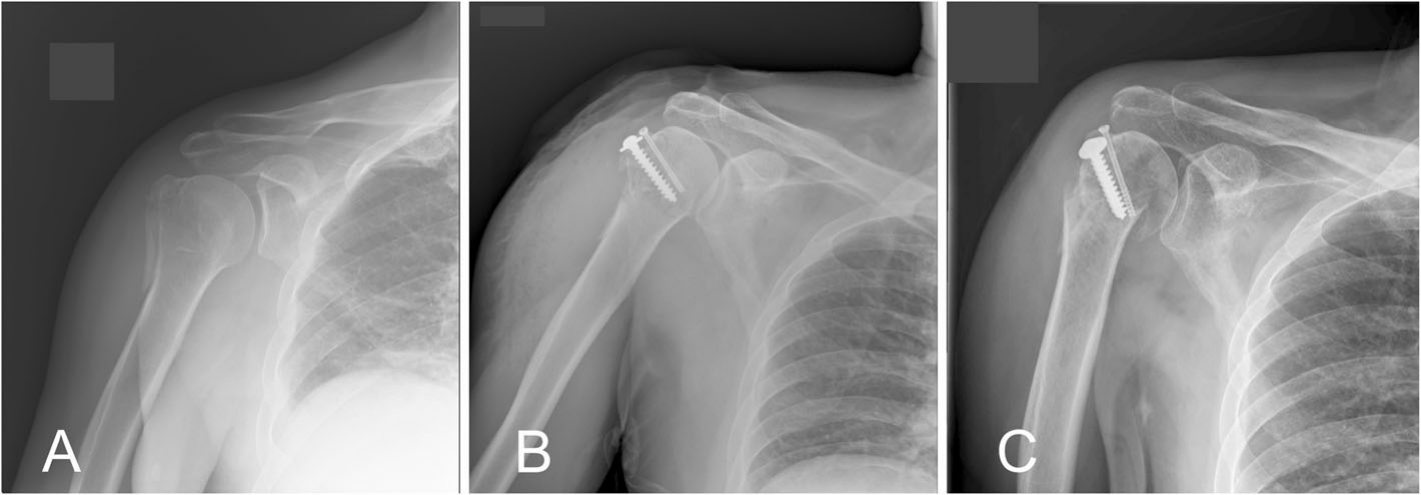
Varus angulation of the humeral surgical neck fracture in a patient who received a screw fixation for the GT fracture. Radiographs illustrated in (**a**) preoperative day; (**b**) postoperative day 1; and (**c**) postoperative day 81

Occult surgical neck fractures were found in 5 out of 68 (7.4%) patients, whereas missed surgical neck fractures were found in 3 out of 68 (4.4%) patients. Of these 8 patients, two were treated non-operatively, while six were fixed surgically for the GT fracture. During the follow-up, one of the 8 patients developed varus angulation, while the seven remaining patients did not have significant displacement or angulation of the surgical neck fracture. None of the 8 patients obtained further surgery. In the patient with the varus angulation of surgical neck fracture, subtle fracture was noted after performing the screw fixation for the GT fracture; thereafter, a head-shaft angle greater than 30° was observed to have developed in the late follow-up radiographs (Fig. 4).

The evidence of occult surgical neck fractures were detected at an average of 18.5 days (4–52 days) after the initial visit to the Emergency Department. In two of these patients, evidence of new bone formation was detected in follow-up radiographs, implying the presence of an occult fracture. The demographic data, GT fracture patterns, and treatment methods of these patients are shown in Table 1.

**Table 1:** Demographic data, fracture pattern of greater tuberosity, the day when the retrospective diagnosis of surgical neck fracture was made, and the deformity of the surgical neck fracture after follow-up for occult and missed surgical neck fractures

Patient No.	Age range	Cause of injury	Fracture classification	Treatment	Day elapsed until the diagnosis	Deformity of the surgical neck	Occult or missed fracture
1	30–40	High energy injury	Split-type	Screw	1	Varus	Occult
2	40–50	Low energy injury	Split-type	Screw	–	Negative	Missed
3	40–50	High energy injury	Split-type	Screw	–	Negative	Missed
4	50–60	Low energy injury	Split-type	Screw	–	Negative	Missed
5	20–30	High energy injury	Split-type	Screw	4	Negative	Occult
6	50–60	High energy injury	Split-type	Screw	5	Negative	Occult
7	40–50	Low energy injury	Avulsion	Non-operative	13	Negative	Occult
8	20–30	High energy injury	Split-type	Non-operative	52	Negative	Occult

Of the 68 patients, 32 had the split-type GT fracture, of which seven (21.9%) also had occult surgical neck fractures. Meanwhile, 32 patients had the avulsion-type GT fracture, of which only one (3.1%) had occult surgical neck fracture. The remaining four patients were classified as having the depression-type GT fracture, none of which had occult surgical neck fracture. Although the proportion of occult surgical neck fracture was higher in the split-type GT fracture (21.9%) than in the avulsion-type GT fracture (3.1%), the difference was not statistically significant (*p* = 0.056).

The inter-rater ICC for classification of GT fractures was 0.8, which corresponds to excellent agreement. The inter-rater ICC for detection of surgical neck fracture range from 0.58 to 0.77 and intra-rater ICC ranged from 0.65 to 0.68 corresponding to good agreement. (Table 2).

**Table 2:** Interobserver agreement between three senior orthopedic surgeons for GT fracture classification and detection of surgical neck fracture

Intra-class correlation coefficient (ICC)	GT fracture classification	Detection of surgical neck fracture	
Inter-rater ICC	0.8	0.58 ~ 0.77	
Intra-rater ICC		0.65 ~ 0.68	

ICC rating: Excellent-Between 0.75 and 1.00, Good-Between 0.60 and 0.74, Fair-Between 0.40 and 0.59, Poor-Less than 0.40

## Discussions

The present study evaluated the occurrence of occult surgical neck fractures in isolated GT fracture, the incidence of which was 7.4% (5 of 68), whereas 4.4% (3 of 68) missed surgical neck fractures were found. One of these 8 patients developed varus angulation, while the other seven patients did not have significant displacement or angulation of the surgical neck during follow-ups. Given that the presence of a surgical neck fracture would significantly affect the treatment strategy, more information about the manifestation of occult surgical neck fracture would certainly help surgeons in managing patients diagnosed with isolated GT fracture.

A surgical neck fracture without appropriate treatment may lead to varus deformity or fracture-displacement of the proximal humerus [8–10]. In Hauschild et al., patients with surgical neck fractures that were treated non-operatively were more likely to have a greater degree of varus deformity [5]; moreover, patients with operative treatment had less coronal plane malalignment and better improvement of pain 3 months after surgery [5]. Court-Brown et al. also reported the outcomes of 99 patients with impacted varus-type surgical neck fractures who were treated non-operatively, and found that 59% of these patients had varus angulation greater than 10° [9]. In the current study, only one patient who received the screw fixation for GT fracture had a varus deformity more than 30° at the humeral surgical neck during follow up. This may suggest that the integrity of occult surgical neck fracture could be sufficiently maintained during follow-ups, unless vigorous manipulation was applied during the operation, in which case displacement or angulation of the fracture may occur. A recent study published by Handoll et al. concluded that there were no clinically significant differences between operative and non-operative treatment in proximal humeral fracture involving the surgical neck [11]. Be that as it may, patients with missed or delayed diagnosis of surgical neck fractures in GT fractures would receive improper stabilization and rehabilitation protocols, possibly leading to substantial sequelae due to insufficient immobilization. Early detection of humeral surgical neck fracture may assist surgeons in proposing an appropriate treatment strategy.

Moreover, the prevalence of occult surgical neck fracture associated with GT fracture is an important issue for discussion. In our study, the radiographs performed during initial ER visit showed a subtle fracture in 3 patients, which could be considered as neglected surgical neck fractures from the initial radiographs after re-evaluation. Thus, the surgical neck fracture was neglected in about 4.4% (3/68) of patients diagnosed with isolated GT fractures. Unfortunately, this figure appears embarrassing for colleagues who initially excluded fracture at the ER; however, one possible explanation is that in our study we asked the orthopedic doctors who reviewed the images to focus on the presence of the likely fracture of the proximal humerus carefully, and so the neglected surgical neck fracture could therefore be found. We believe that the high rate depends on our awareness of the final diagnosis and, perhaps, to our particular experience in this field. Another potential explanation for the misdiagnosed fractures of the surgical neck may be attributed to suboptimal radiographs examination. In most of our patients, only the anteroposterior in internal and external rotation views were performed as routine evaluation in our hospital since there was no evidence of surgical neck fracture, and so the lack of scapular and axillary views may interfere with an accurate diagnosis. Moreover, we believe that raising awareness of the existence of occult surgical neck fracture would help physicians avoid making such misdiagnoses. We also suggest physicians to inspect the surgical neck carefully while evaluating patients with isolated GT fractures. Further, computed tomography (CT) imaging could be considered if a suspect lesion is found.

Although not statistically different, the proportion of occult surgical neck fracture was higher in the split-type GT fracture (21.9%) than in the avulsion-type GT fracture (3.1%) in our study. As aforementioned, Mutch et al. proposed GT fracture classifications, and also reported that the distribution of avulsion-, split- and depression-type fractures was 39, 41, and 20%, respectively [7]. For the patients with isolated GT fracture in our study, 32 (47%) had the split-type, 32 (47%) had the avulsion-type, and the remaining 4 (6%) had the depressed-type GT fracture. Although there were fewer patients that had the depression-type GT fracture in this study, the proportions of the split-type and avulsion-type GT fractures were quite similar to Mutch et al. who also reported that GT fracture occurs as the result of different trauma mechanisms [7]. A split-type fracture involves a large fragment with a vertical fracture line, and is thought to be caused by impaction on the anterior surface of the glenoid during dislocation or subluxation of the shoulder, while the mechanism of an avulsion fracture is similar to that which causes a rotator cuff tear [7]. Since occult surgical neck fracture occurred more frequently in the split-type GT fracture, we believe that the trauma mechanism may account for this phenomenon. Accordingly, clinical physicians should be especially aware of the occult surgical neck fracture when treating split-type GT fractures.

### Limitations

There are some limitations in this study. First, the retrospective collection of data included a wide range in both follow-up duration and the heterogeneity of the patient population; also, not all patients had standardized measurement of functional outcome way. Therefore, limited information was provided regarding the outcomes of the patients with GT fracture and missed or occult surgical neck fracture in this study. Second, isolated GT fracture is not a common fracture, and we were only able to gather a small cohort with few cases of occult fractures over a 13-year period in a large scale medical center, thereby limiting our ability to discover possible risk factors of occult surgical neck fracture. As such, we believe that further studies are needed to better understand if GT fracture caused by different intensities of trauma may be associated with subtle surgical neck fractures that may be erroneously interpreted as intact at the initial radiograph. Third, the views in radiographs for our patients were only anteroposterior views of internal and external rotation of the proximal humerus. Actually, the lack of sufficient views in radiographs during initial and follow-up evaluation would possibly affect the clinical judgments. We suggested taking more views of radiograph once there is a suspicious surgical neck fracture. Moreover, the reported rate may be underestimated because such occult surgical neck fractures may remain undiagnosed in patients who did not later obtain follow-up radiograph examinations. In addition, some patients’ occult fractures might have been missed at our hospitals and diagnosed as having occult fractures at other hospitals. Be that as it may, information regarding the clinical outcomes of these patients is unknown. Further, we could not analyze the mechanism of injury between different groups due to incomplete documentation in this retrospective review. It is also possible that more sensitive radiographic studies, such as magnetic resonance imaging or bone scan, may have detected a higher incidence of occult surgical neck fractures in these patients.

## Conclusion

Occult humeral surgical neck fractures occurred in 7.4% of isolated greater tuberosity fractures after re-evaluation, while missed humeral surgical neck fractures occurred in 4.4%. We suggest physicians to inspect the surgical neck carefully while evaluating patients with isolated GT fractures, especially the split-type GT fracture.

### Clinical relevance

A delayed diagnosis of occult surgical neck fracture might result in further displacement, subsequently leading to increased morbidity.

## Data Availability

The datasets used and/or analyzed during the current study are available from the corresponding author on reasonable request.

## Abbreviations

GT: greater tuberosity
ICC: intra-class correlation coefficient
